# HER2 copy number determination in breast cancer using the highly sensitive droplet digital PCR method

**DOI:** 10.1007/s00428-023-03706-3

**Published:** 2023-11-23

**Authors:** Beate Alinger-Scharinger, Cornelia Kronberger, Georg Hutarew, Wolfgang Hitzl, Roland Reitsamer, Klaassen-Federspiel Frederike, Martina Hager, Thorsten Fischer, Karl Sotlar, Heidi Jaksch-Bogensperger

**Affiliations:** 1 Department of Pathology, University Hospital and Paracelsus Medical University Salzburg, Muellner Hauptstraße 48, 5020 Salzburg, Austria; 2 Research Management and Technology Transfer, Paracelsus Medical University Salzburg, Strubergasse 16, 5020 Salzburg, Austria; 3 Department of Ophthalmology and Optometry, Paracelsus Medical University Salzburg, Muellner Hauptstraße 48, 5020 Salzburg, Austria; 4 Research Program Experimental Ophthalmology and Glaucoma Research, Paracelsus Medical University Salzburg, Muellner Hauptstraße 48, 5020 Salzburg, Austria; 5 Department of Obstetrics and Gynaecology, Clinical Research Center Salzburg (CRCS), University Hospital and Paracelsus Medical University Salzburg, Muellner Hauptstraße 48, 5020 Salzburg, Austria

**Keywords:** Breast cancer, HER2, ERBB2, Droplet digital PCR (ddPCR), Copy number

## Abstract

Human epidermal growth factor receptor 2 (HER)-positive breast cancer (BC) is characterized by an aggressive clinical course. In the case of HER2 overexpression/amplification, patients benefit from HER2-targeting therapies. Standardized diagnostic HER2 assessment includes immunohistochemistry (IHC) and/or in situ hybridization (ISH). The aim of this study was to compare this “gold standard” with the Droplet Digital™ polymerase chain reaction (ddPCR), a method that allows sensitive and precise detection of copy number variations (CNV) in FFPE (formalin-fixed, paraffin-embedded) DNA samples. Partitioning of the PCR reaction into 20,000 droplets enables a precise quantitative “CN” discrimination also in heterogeneous samples. FFPE breast cancer samples (*n* = 170) with routinely assessed HER2 status by IHC/ISH were retrospectively analyzed using the *ddPCR CNV ERBB2 assay*. Comparison of HER2 status assessment by the two methods revealed concordant results in 92.9% (158/170) of the cases. Discrepant cases were verified and interpreted. For ddPCR, a cut off value of 3 HER2 copies was set to distinguish between HER2-negative and HER2-positive BC. Results obtained with the *ddPCR CNV ERBB2 assay* were consistent and reproducible, and serial dilutions demonstrated a high stability and sensitivity of the method. The *ddPCR CNV ERBB2 assay* may be a specific and convenient tool to quantify HER2 copy numbers in BC samples. In our study, this method showed high reproducibility in accuracy of HER2 assessment compared to IHC/ISH analysis.

## Introduction

Breast cancer (BC) is a leading cause of cancer death in women. Patient survival highly depends on tumor stage at diagnosis and tumor biology. Beside estrogen and progesterone receptor (ER/PR) status of the human epidermal growth factor receptor 2 (HER2, *ERBB2*) is an important prognostic and predictive biomarker and is strongly expressed in 15–20% of newly diagnosed invasive BCs (IBC) [1–4]. HER2 overexpression caused by gene amplification leads to ligand-independent activation through receptor dimerization, subsequent transphosphorylation of their intracellular domains, and further tyrosine kinase-mediated activation. The consequence is uncontrolled proliferation and tumor formation [5–7]. HER2-positive BCs show an adverse prognosis with increased mortality rates in early-stage disease as compared to HER2-negative (ER/PR-positive) BCs, reduced time to relapse, and increased incidence of metastases especially to the brain [8]. Patients benefit from HER2-directed treatments in the adjuvant and/or neoadjuvant setting [9–12]. According to recommendations of the American Society of Clinical Oncology/College of American Pathologists (ASCO/CAP), HER2 expression in primary BC is usually assessed by immunohistochemistry (IHC) with concluding classification into HER2 positive (IHC 3 +), HER2 equivocal (IHC 2 +), or HER2 negative (IHC 1 + or 0) [13–16]. Biopsy samples with equivocal HER2 status are additionally assessed via in situ hybridization (ISH) to confirm or exclude HER2 gene amplification. Several commercially available testing kits have received approval from the FDA for the assessment of HER2 status [17]. Though standardized assays for IHC and ISH are used, current HER2 testing methods show an inaccuracy in up to 20% of cases. This refers to pre-analytical and test performance parameters [18, 19] as well as to interpretation of IHC and/or ISH results by the pathologist [14]. Especially cases like BC with genetic heterogeneity and chromosome 17 aneusomy have been identified as diagnostic challenges [15]. Interestingly, in HER2-positive patients, precise determination of *ERBB2* copy numbers may be of predictive value since patients with a so-called high-level amplification have been shown to be more responsive to HER2-targeting therapies [20–22]. The Droplet Digital™ PCR (ddPCR) is a sensitive method to accurately define *ERBB2* copy numbers in FFPE samples of BC patients [23]. It is an efficient and reliable method with a high throughput that can be adapted for a wide variety of applications using specially designed and validated PrimePCR™ Assays. To receive the high sensitivity and specificity of the method, the PCR reaction is massively partitioned creating 20,000 droplets allowing measurement of thousands of independent amplification events within a single sample. The aim of the study was to evaluate the *CNV ERBB2 assay* from Bio-Rad as an additional screening tool in HER2 diagnostic and to examine whether HER2 positivity can be correlated with ddPCR copy number calculation.

## Material and methods

### Patient cohort

A cohort of FFPE BC biopsies (*n* = 170) with at least 30% histologically confirmed tumor cell content was re-investigated for its HER2 status to establish the *ddPCR CNV ERBB2 assay* (Bio-Rad, Hercules, CA, USA). Inclusion criterion was a known HER2 status, determined during routine diagnostics, independent of women’s age, tumor classification, tumor size, nodal status, and ER, PR, or Ki67 values (Table 1). The cohort included 3 groups: (1) HER2 negative (0/1 + ; *n* = 82), (2) HER2 equivocal (2 + ; *n* = 52) with positive amplification result by ISH (*n* = 16) and with negative amplification result by ISH (*n* = 36), and (3) HER2 positive (3 + ; *n* = 36) cases (Table 1).

**Table 1:** Summary of FFPE breast cancer samples used for establishment of the *ddPCR CNV ERBB2 assay* including distribution of HER2 groups

	HER2 IHC-defined groups			Total (%)
	IHC 0/1 + (%)	IHC 2 + (%)	IHC 3 + (%)	
Total number, *n* (%)	82 (48.2)	52 (30.6)	36 (21.2)	170 (100)
Histological grade				
1	17 (10)	4 (2.4)	2 (1.2)	23 (13.5)
2	26 (15.3)	37 (21.8)	22 (12.9)	85 (50)
3	39 (22.9)	11 (6.5)	12 (7.1)	62 (36.5)
Hormone receptor (ER/PR)				
Negative	40 (23.5)	5 (2.9)	17 (10)	62 (36.5)
Positive	42 (24.7)	47 (27.6)	19 (11.2)	108 (63.5)
Ki-67 index				
≥ 20%	53 (31.2)	28 (16.5)	31 (18.2)	112 (65.9)
< 20%	29 (17.1)	24 (14.1)	5 (2.9)	58 (34.1)

### Immunohistochemistry and in situ hybridization

IHC staining and additional ISH of HER2 2 + positive samples were performed during routine diagnostic evaluations at the Department of Pathology, University Hospital and Paracelsus Medical University Salzburg, using standardized operation procedures. Scoring and interpretation of the results were performed by experienced breast pathologists according to ASCO/CAP guidelines [14]. The tissue samples were routinely processed, including formalin-fixation and paraffin-embedding (FFPE). Immunohistochemical staining was performed fully automated on a Dako Omnis Immunostainer (Agilent, Santa Clara, CA, USA) using the *polyclonal Rabbit Anti-Human c-erbB-2 Oncoprotein* antibody (1:600, Agilent, Santa Clara, CA, USA) [24]. For silver-enhanced in situ hybridization (SISH), the *Ventana®* HER2 *Dual ISH DNA Probe cocktail* (Roche, Basel, Switzerland) was applied on the VENTANA BenchMark ULTRA System (Roche, Basel, Switzerland) as described by Lim et al. [25].

### Droplet digital PCR CNV ERBB2 assay

We used the *Maxwell® RSC DNA FFPE Kit* (Promega, Madison, WI, USA) with Maxwell® RSC Instruments for automated purification of the genomic DNA from FFPE tissue samples. DNA quantification was performed using the Quantus™ Fluorometer together with *QuantiFluor® Dye System* (Promega, Madison, WI, USA).

For copy number analysis, we used the *ddPCR CNV ERBB2 assay* (Bio-Rad, Hercules, CA, USA) and *WSB1* as standard reference gene for its genetic location close to centromere region of chromosome 17. Both are 20 × concentrated, ready-to-use primer–probe mixes optimized for use with *ddPCR Supermix for Probes (no dUTP)*. The two probes are labeled with the fluorophores FAM (*ERBB2*) and HEX (*WSB1*). Mastermix for ddPCR was set up as recommended in the instructor’s manual. We performed validation steps for DNA concentration and enzyme digestion. Droplet generation was performed in the QX-200 Droplet Generator (Bio-Rad, Hercules, CA, USA). The oil-PCR reaction mixture was transferred to a ddPCR 96-well plate, and PCR was performed in a Bio-Rad cycler with a deep-well block. PCR conditions were as follows: 10 min at 95 °C for enzyme activation, 40 cycles of 30 s at 94 °C for denaturation and 1 min at 60 °C for annealing/extension, 10 min at 98 °C for enzyme deactivation, and final hold at 10 °C. After PCR cycling, the plate was placed into a QX-200 Droplet Reader for signal counting.

Results for copy number (CN) data were analyzed using the QuantaSoft software (Bio-Rad, Hercules, CA, USA) by calculating the ratio of *ERBB2* concentration to *WSB1* concentration. As the centromeric region of chr17 where *WSB1* is located may be amplified, this in fact is not by definition a true *ERBB2* copy number but a ratio, as in many dual-FISH tests. Since this commercial software refers to the end result as CN, we prefer to follow this but further refer to this as “CN.”

**Fig. 1:**
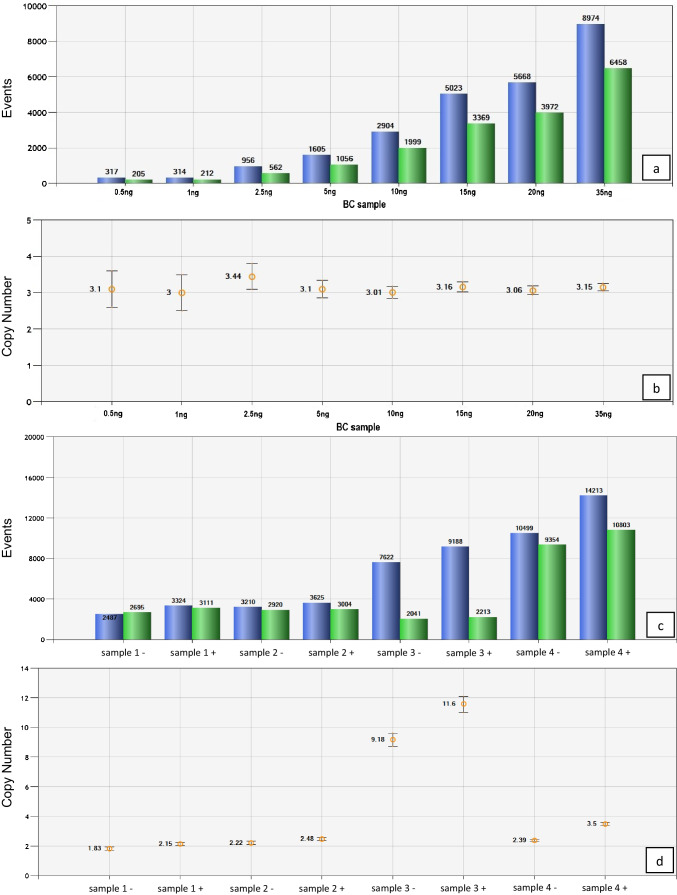
Adjustment of DNA concentration and enzyme digestion to the basic Droplet Digital™ polymerase chain reaction protocol. **a, c** The droplet counts (*ERBB2*: blue bars; *WSB1*: green bars) and **b, d** the corresponding copy number calculation. **a** A serial dilution of a HER2-positive breast cancer sample with a range from 0.5 to 35 ng DNA in 8 dilution steps. Copy number is comparable for **b** all concentrations. **c** Four samples performed with (all samples with +) and without *HaeIII* digestion (all samples with −). Difference between *ERBB2* (blue bars) and *WSB1* (green bars) droplets was higher with digestion, leading to a change in **d** copy number ratio. For sample 4 the interpretation of the result changes from negative to positive as with digestion the copy number elevates from 2.39 to 3.5 and therefore over the threshold of 3 (**d**, sample 4 +). The error bars represent a 95% confidence interval

To define DNA concentration used for ddPCR analysis and additionally to demonstrate sensitivity of the method, a serial dilution of a small test cohort of *n* = 5 HER2-positive BC samples was performed using 8 dilution steps: 35 ng, 20 ng, 15 ng, 10 ng, 5 ng, 2.5 ng, 1 ng, and 0.5 ng (Fig. 1a, b). Finally, we used a concentration of 30 ng of DNA in our setup; each sample was investigated in duplicate.

To separate closely linked *ERBB2* copies [4] in case of a HER2 gene amplification, DNA of the test cohort (*n* = 5) was digested with restriction enzyme *HaeIII* (New England BioLabs, Ipswich, MA, USA). Two modes of digestion were performed and compared for their diagnostic utility and convenience. First, 10 units of the restriction enzyme *HaeIII* were directly added to the PCR master mix and incubated for 15 min at room temperature. Second, sample DNA was digested with *HaeIII* for 1 h at 37 °C before merging with the PCR master mix. In addition, *n* = 97 samples were investigated with and without *HaeIII* digestion to further address the necessity of the digestion step.

### Statistical methods

Data were checked for consistency using McNemar’s test to analyze cross tabulations and to compare probabilities for HER2-positive cases assessed by both methods. Hodge-Lehman confidence intervals were computed for difference of both probabilities and Pearson-Clopper confidence intervals for sensitivity, specificity, negative, and positive predictive values. An univariate logistic regression model was applied to the relation between ddPCR *ERBB2* copy number and risk for IHC/ISH positive HER2 status. All reported tests were two-sided, and *p*-values < 0.05 were considered statistically significant. All statistical analyses in this report were performed with the software tool STATISTICA 13 [26].

## Results

### DNA concentration validation

To determine the minimal and optimal amount of input DNA, serial dilutions to 35 ng, 20 ng, 15 ng, 10 ng, 5 ng, 2.5 ng, 1 ng, and 0.5 ng of input DNA of HER2-positive samples (*n* = 5) were investigated in duplicates. Even with the lowest amount of 0.5 ng of input DNA copy number calculations were successful and showed consistent results for HER2 “CN” calculations throughout the various dilution steps in each case, thus demonstrating the high sensitivity and stability of the method (Fig. 1a, b).

### HaeIII digestion protocol

Comparison of two modes of *HaeIII* digestion, (1) co-incubation of digestion enzyme and PCR mix including sample DNA for 15 min at RT versus (2) predigestion of *HaeIII* and sample DNA for 1 h at 37 °C, showed equal CNV results for all 5 investigated test cases (data not shown). We therefore decided to use mode 1 (co-incubation of digestion enzyme and PCR mix including sample DNA for 15 min at RT) because of time reduction.

### Necessity of HaeIII enzyme digestion

In *n* = 97, BC samples we compared ddPCR with and without *HaeIII* digestion to verify the necessity of the *HaeIII* digestion step for separating closely linked *ERBB2* copies [4] in HER2 amplified cases. Performance without *HaeIII* digestion step showed considerably reduced copy number values compared to performance with digestion (Fig. 1c, d). Using a cut-off value of 3.0 (see following chapter), we identified 28 cases (28.9%) as HER2 positive when the digestion step was performed, but only 18 cases (18.6%) were above the cut-off level of 3.0 without the digestion step. This clearly illustrates the necessity of the digestion step in the ddPCR protocol, as 10 cases (35.7%) would have been assessed as HER2 negative without *HaeIII* digestion. Consequently, subsequent statistics only implied “CN” values of ddPCR protocol with digestion step.

### Determination of cut-off value for HER2-positive cases

**Fig. 2:**
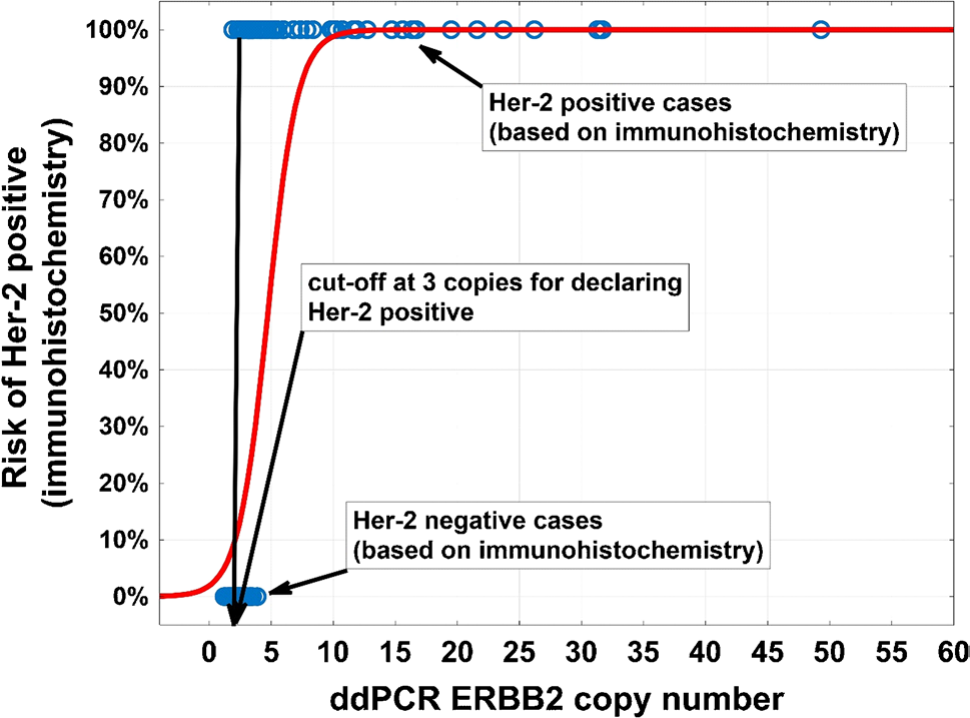
Logistic regression model of risk for HER2 positive (based on immunohistochemistry) depending on ddPCR *ERBB2* copy number. The cut-off was set at 3 copies for declaring HER2 positive with ddPCR

After investigation of all 170 BC samples using the *ddPCR CNV ERBB2 assay*, we verified the cut off value for HER2-positive interpretation of ddPCR results. Based on logistic regression model (Fig. 2) comparing IHC/ISH and ddPCR data, a cut-off value of 3.0 was set. Based on known HER2 IHC scores and, if relevant, ISH results, mean ddPCR “CN” values were calculated, being 2.3 for IHC 0, 2.2 for IHC 1 + , 2.2 for IHC 2 + /ISH − , 4.9 for IHC 2 + /ISH + , and 13.3 for IHC 3 + tumors, respectively. Notably, the set cut-off value of 3.0 for ddPCR is different from a cut-off defined in the 2018 ASCO/CAP guidelines for ISH analyses, where a tumor is defined positive with a HER2/CEP17 ratio above 2.0, provided the mean HER2 copy number is above 4 per diploid genome.

### HER2 scoring based on ddPCR CNV determination

The 170 BC samples were classified as positive or negative based on their ddPCR “CN” values and compared with the primary HER2 classification by IHC/ISH established during routine processing. In Table 2, the comparison between IHC/ISH and ddPCR is shown. In total, IHC/ISH identified 53/170 (31.2%) patients with a positive HER2 status while ddPCR (> ddPCR *ERBB2* copy number above 3.0) detected 51/170 (30.0%), respectively. No significant difference was found between both proportions (McNemar test, two-sided, *p* = 0.77) demonstrating the accuracy of the *ddPCR CNV ERBB2 assay*. Concordant results between IHC/ISH and ddPCR were obtained in 92.9% of the patients (158/170; 95% CI, 88–96.3; Table 2) with double-positive results in 65.9% (112/170) and double-negative results in 27.1% (46/170) of the patients. Specificity and sensitivity for ddPCR were 95.7% (95% CI, 90.3–98.6) and 86.8% (74.7–94.5). Negative and positive predictive values were 94.1% (95% CI, 88.3–97.6) and 90.2% (78.6–96.7) compared to IHC/ISH results.

**Table 2:** Correlation between immunohistochemistry and ddPCR including specificity and sensitivity. No significant difference was found when comparing both proportions (McNemar test, two-sided, *p* = 0.77) before and after re-evaluation of discrepant cases

HER2 status (IHC/ISH)	ddPCR ERBB2 copy number < 3	ddPCR ERBB2 copy number ≥ 3	Total number
Results before re-evaluation; concordance 92.9%			
Negative (%)	112 (65.9)	5 (2.9)	117 (68.8)
Positive (%)	7 (4.1)	46 (27.1)	53 (31.2)
Results after re-evaluation; concordance 96.5%			
Negative (%)	114 (67.1)	1 (0.5)	115 (67.6)
Positive (%)	5 (2.9)	50 (29.4)	55 (32.4)
Totals (%)	119 (70)	51 (30)	170 (100)

### Re-evaluation of tumors with discrepant results

**Fig. 3:**
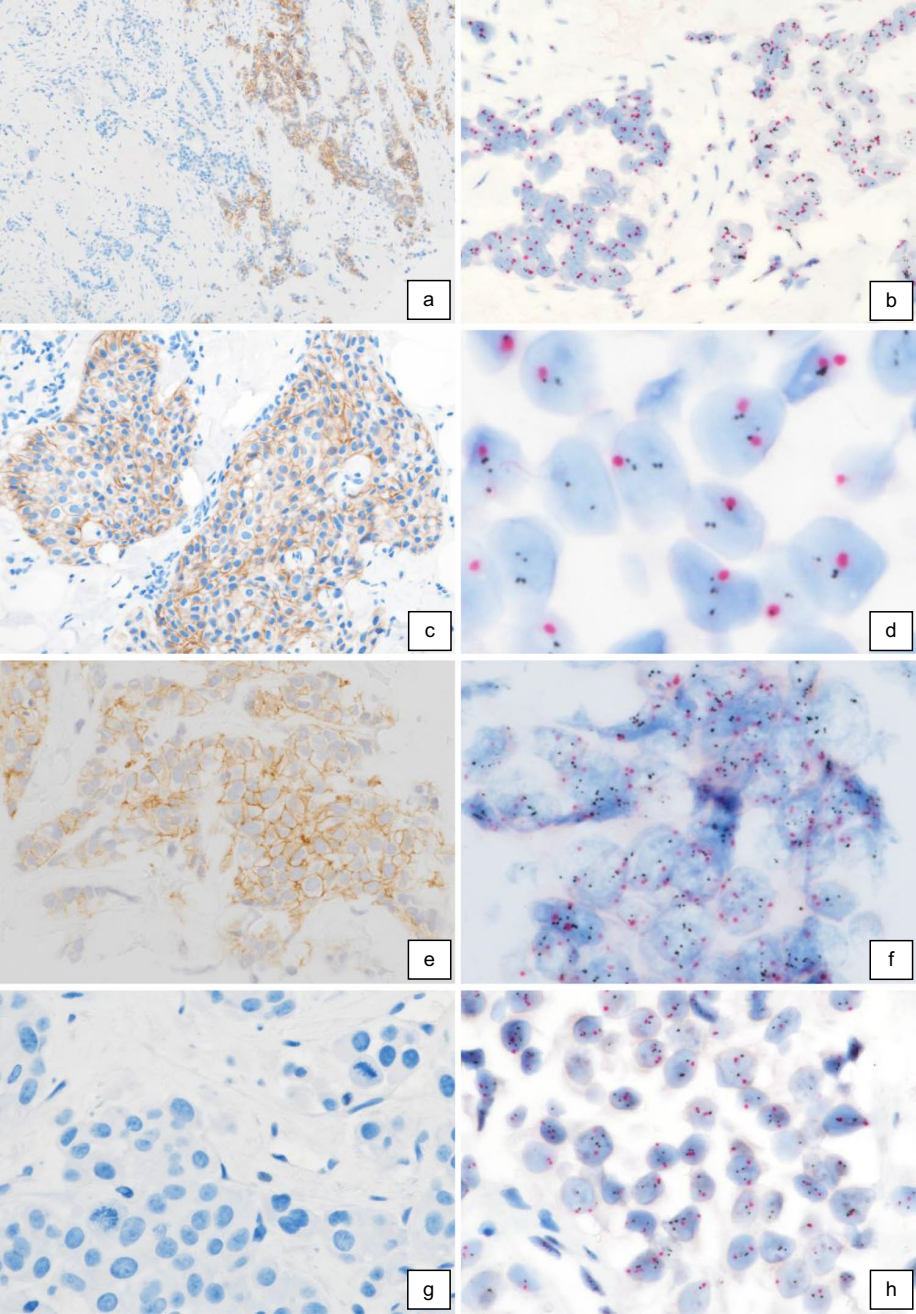

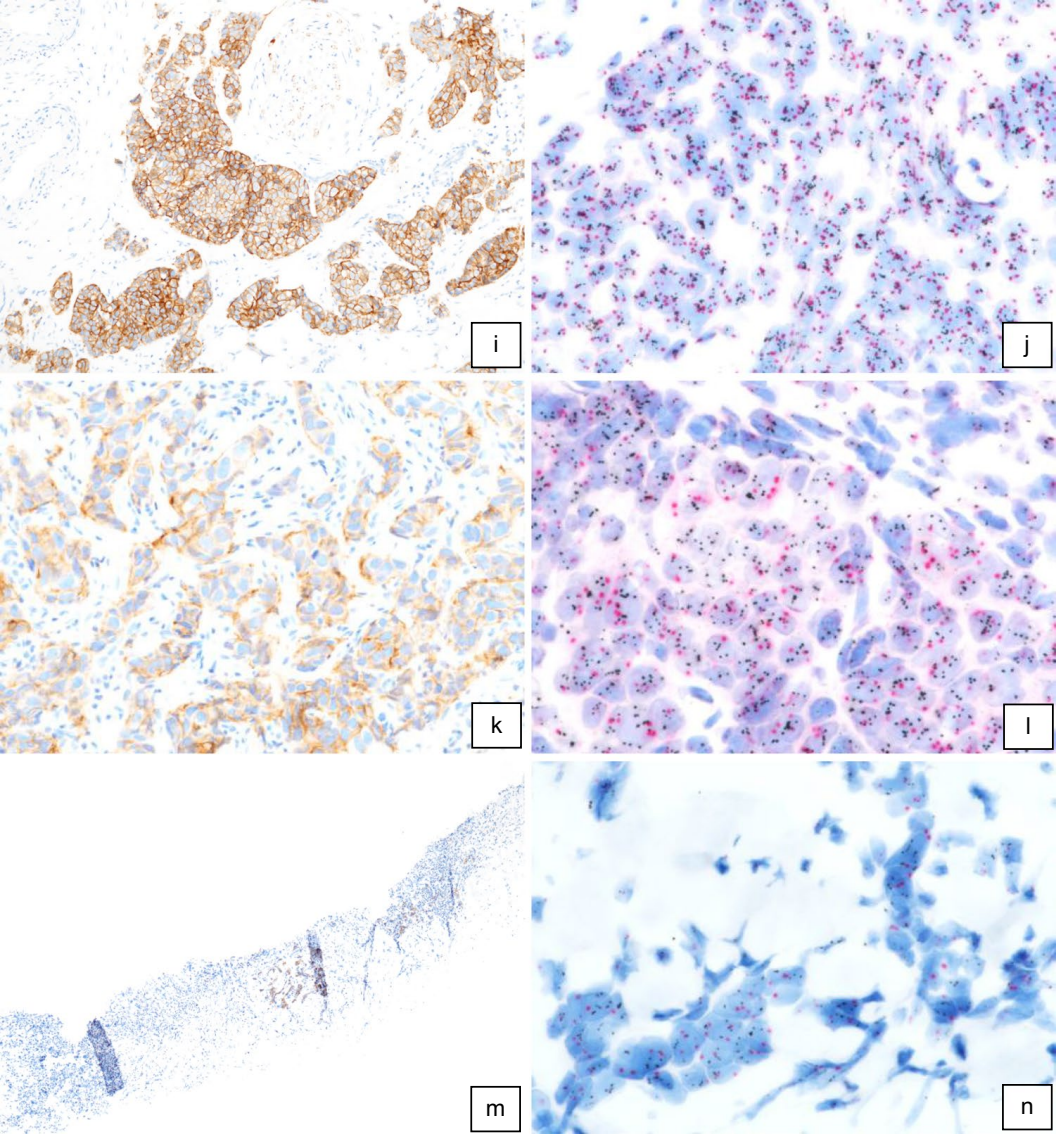
**IHC and ISH results in cases with discrepant histomorphologic and ddPCR findings. **Heterogeneous Her2 expression (**case 12**) in about 10% of tumor cells (**a**, 100x). Tumor areas with Her2 3+ expression showed clear-cut Her2 amplification indicated by multiple Her2 gene copies (small black dots) per nucleus and a Her2/CEP17 (larger red dots) ration of 3.6 (**b**, 400x). Invasive BC with solid growth pattern (**case 9**) and inconclusive Her 2 expression (**c**, IHC 2+, 200x). Tumor cells showed CEP17 monosomy and <4 Her2 copies per tumor cell (**d**, 630x). Her2/CEP17 ratio accounted for 1.9. The case also showed a borderline-negative result by ddPCR. Inconclusive IHC Her2 2+ score (**e**, 200x; **case 5**) with positve ddPCR results correctly diagnosed with Her2 amplification during reevaluation (**f**, 400x). A ddPCR-positive case without Her2 expression (**g**, 400x; IHC 0, **case 1**) and without signs of Her2 amplification (**h**, 400x). Another ddPCR-positive tumor initially scored IHC 1+ (**case 4**). During reevaluation an IHC Her2 2+/3+ score was established (**i**, 100x) and Her2 amplification could be demonstrated by ISH (**j**, 400x). Inconclusive Her2 2+ expression (**k**, 200x; **case 7**) and a negative ddPCR result in a patient with CEP17 **polysomy** showing a positive ISH result based on a Her2/CEP17 ration of 3.1 and a Her2 copy number of 6.3 per cell (l, 400x). Heterogeneity with Her2 overexpression (**m**, 50x; **case 2**) and amplification in only a small tumor area (N, 400x) resulted in false-negative ddPCR

Discordant results between IHC/ISH and ddPCR were found in 7.1% (12/170) of our cases (Table 2) with 7 cases of negative ddPCR results in patients with IHC/ISH-positive tumors and with 5 cases of positive ddPCR results but a negative IHC/ISH status (Table 2). All these cases were re-investigated with both ddPCR and IHC/ISH (Table 3). For ddPCR, we re-used the same DNA, IHC and ISH stainings were freshly prepared and evaluated. The obtained values for *ERBB2* “CNs” determined by ddPCR were reproducible and showed no discrepancies compared with initial results. Re-evaluation of IHCs and/or ISHs revealed some changes in its final scores and, more importantly, final assessment as positive or negative. In cases 3 and 4 with negative primary IHC interpretation (IHC 1 +) but positive ddPCR results, re-evaluation of IHC in both cases in fact revealed an “inconclusive” result (IHC 2 +) in patient 3 and a “positive” result (IHC 3 +) in patient 4 (Fig. 3i). Newly performed SISH preparations revealed positive results with HER2/CEP17 ratios of 2.4 and 5.6 (Fig. 3j), respectively, thus demonstrating concordance of ddPCR and IHC/ISH results. Initially determined SISH results of 1.4 and 1.7 in cases 5 and 6 (Table 3), both with IHC 2 + scores (Fig. 3e, case 5), were found to be false-negative during re-evaluation. The newly determined HER2/CEP17 ratios of 2.3 (Fig. 3f) and 2.8 corresponded well with positive ddPCR results. Cases 10 and 11, initially scored IHC 3 + , were both downgraded during re-evaluation. In case 10, a total of 70% of tumor cells showed a continuous membrane staining but only with moderate intensity, in fact representing an IHC 2 + score. Case 11 had to be downgraded to IHC 1 + after exclusion of a larger DCIS component showing a strong HER2 expression. Likewise, re-evaluated SISH results of 1.2 and 1.5 also did not reach the threshold of 2.0, thus, in concordance with ddPCR results, indicated non-amplified tumors in both cases. Tumor cells in case 9 (Fig. 3c) showed HER2 monosomy (Fig. 3d), a HER2/CEP17 ratio of 2.2 with a median HER2 copy number of < 4, and just a negative ddPCR test of 2.9. According to ASCO/CAP guidelines the SISH preparation was blindly reassessed by two other colleagues and finally determined “negative” (HER2/CEP17 ratio 1.96, Table 3). The remaining cases 1, 2, 7, 8, and 12 still showed discrepant results after re-evaluation, being either negative by ddPCR or by IHC/SISH (Table 2). Two of these tumors (cases 2 and 12) showed remarkable heterogeneity in HER2 expression/amplification by IHC/SISH with clearly amplified tumor areas, but not reaching cut-off levels in ddPCR investigating the whole tumor (case 2 in Fig. 3m, n and case 12 in Fig. 3a, b). Case 7 may be missed by ddPCR because of CEP17 copy number increase (Fig. 3k, l), and case 8 was borderline negative by ddPCR (2.9) and borderline positive by SISH (HER2/CEP17 ratio 2.1). Only case 1 shows clearly discrepant results in ddPCR and IHC/ISH without comprehensible explanation (Fig. 3g, h).

**Table 3:** Summary of the discrepant cases. Re-evaluation of the cases included IHC, ISH and ddPCR. The discrepancies in cases 3–6 and 9–11 were comprehensible after re-evaluation as they occurred because of different interpretation of IHC and/or ISH results. Cases 1, 2, 7, 9, and 12 demonstrate uncommon ISH HER2 groups and intratumoral heterogeneity

Case number	Tumor %	ddPCR ERBB2 copy number	Initial Her2 IHC-defined groups	Initial SISH	Re-evaluation HER2 IHC (positive cells %)	Re-evaluation SISH	Comparison
1	80%	3.5	0	1,2	0 (0)	1.5	Discrepant
2	30%	2.5	0	3,85	2 + (15)	3.6	Discrepant; heterogeneity
3	60%	3.1	1 +		2 + (30)	2.4	Concordant
4	60%	3.9	1 +		3 + (40)	5.6	Concordant
5	50–60%	3.3	2 +	1.4	2 + (40)	2.3	Concordant
6	50–60%	3.3	2 +	1.7	2 + (50)	2.8	Concordant
7	50%	2.4	2 +	2.36	2 + (80)	3.1	Discrepant; CEP17 CN increase
8	30%	2.7	2 +	2.8	2 + (90)	2.1	Discrepant; borderline
9	70–80%	2.9	2 +	7.2	2 + (60)	2.2** (1.9)	Concordant; monosomy
10	50%	1.9	3 +	2.6	2 + (70)	1.2	Concordant
11	80% (50% DCIS included)	2.3	3 +		1 + (30)	1.5	Concordant
12	40–50%	2.8	2 +	3.1	3 + * (< 10%)	3.6	Discrepant; heterogeneity

^*^Focal clear-cut IHC 3 + result

^**^Average HER2 copy number < 4

## Discussion

The aim of our study was to verify the *ddPCR CNV ERBB2 assay* for its reliability to facilitate and accelerate HER2 testing on BC core needle biopsies according to 2018 ASCO/CAP guidelines [14]. A cohort of *n* = 170 FFPE BC samples (Table 1) was analyzed for its ddPCR-determined HER2 status in correlation to IHC/SISH results obtained during routine diagnostics.

Copy number variation (CNV) generally implies an amplification or deletion of specific gene sections. For HER2 diagnostics in IBC, the region of interest is the *ERBB2* gene locus [1, 4, 6]. Routine HER2 assessment is based on IHC and/or ISH interpreted by trained pathologists according to internationally accepted ASCO/CAP guidelines [14]. Protocols and handlings are well established, but BC samples with equivocal IHC or intermediate ISH results need further work-up [27–30]. Since the field of cancer therapy is steadily expanding, methods must be constantly adapted to exactly classify those subgroups of cancer patients having realistic chances to benefit from anti-cancer therapies [20–22].

The ddPCR for HER2 “CN” determination may be an ideal alternative to morphology-based methods like IHC and ISH to overcome interobserver variability and to provide an objective, quantitative and reproducible method for the identification of HER2 amplified cancers. Compared to other molecular techniques like MLPA (multiplex ligation-dependent probe amplification) or NGS (next-generation sequencing), which use reference genes to calculate “CN,” the ddPCR is a fast and easy to handle method, without the need of a predefined test menu and also the use of FFPE samples is no limiting factor. In the present study, input DNA was adjusted to 30 ng, which could easily be extracted from IBCs of all core biopsy specimens investigated.

Analyses of serial dilutions of input DNA demonstrated sensitivity and reproducibility of results over a wide range of input DNA ranging down to 0.5 ng, thus ensuring valid results also with minute amounts of tumor tissues. Before amplification, the method requires an enzyme digestion step to separate closely linked DNA copies in cases of HER2 amplification and to allow partitioning of single HER2 DNA copies into amplification droplets. Without the *HaeIII* digestion step, about 36% of cases, found to be amplified after digestion, would have otherwise been missed.

The threshold of ≥ 3.0 for HER2 amplified cases was calculated by univariate logistic regression model, representing the required number of *ERBB2* copies by ddPCR delivering a positive HER2 status in IHC/ISH-positive cases as determined as part of the primary diagnosis. Mean ddPCR values for non-amplified HER2 cases, i.e., IHC 0/1 + and IHC2 + /ISH − , were almost identical, accounting for 2.2 to 2.3. In contrast, they were more than twice as high for IHC 2 + /IHC + and almost six times as high for IHC 3 + cases, accounting for 4.9 and 13.3. Thus, ddPCR may especially be helpful in borderline cases, adding additional information in favor or against anti-HER2 directed therapies and may easily identify those IHC 3 + patients with high-level HER2 amplifications who have significantly higher chances for achieving pathological complete remissions after anti-HER2 treatment alone [8, 20].

In a retrospective study of 170 BC samples comparing routine IHC/ISH results as the “gold standard” with ddPCR identified 12 (7.1%) discrepant cases, 7 of which with positive IHC/ISH and negative ddPCR results and 5 vice versa. Careful re-evaluation of discrepant cases revealed misinterpretation of IHC/ISH results in 6 patients as the main reason for discrepancy. Either cases were underscored as IHC 1 + , preventing ISH preparations which then confirmed HER2 amplifications (cases 3 and 4), or positive ISH results were underscored (cases 5 and 6). Two IHC 3 + cases were overrated, one because of a predominant IHC 3 + DCIS component but the IBC being IHC 1 + and ISH negative (case 11), and the other case in fact being IHC 2 + /ISH − (case 10). The main reason for false-negative ddPCR results were found to be tumor heterogeneity with focal HER2 amplification (cases 2 and 12) and chromosome 17 gains or losses, i.e., CEP17 copy number increase (case 7) or monosomy (case 9). The therapeutical benefit of targeted therapies for these types of BCs is still unclear and under investigation [27, 28, 30]. In addition, a low tumor content most probably resulted in false-negative ddPCR results in case 8. Only in a single patient (case 1) clearly discrepant results with positive ddPCR status and clearly negative IHC/ISH status finally could not be explained satisfactory.

In conclusion, the applied *ddPCR CNV ERBB2 assay* demonstrated to be a robust method for the application in FFPE tissues. It is a high-throughput method for absolute quantitation of HER2 ‘CN’ with high sensitivity and specificity. Before and after re-evaluation of initially discrepant cases comparison of ddPCR with the “gold standard” IHC/ISH revealed concordant results in 92.9% (158/170) and 96.5% (164/170) and negative predictive values of 94.1% and 95.8% (Table 2), thus underlining the high diagnostic reliability. A big advantage of ddPCR-based HER2 CNV determination is interobserver variability, while tumor heterogeneity and chromosome 17 gains and losses are its limiting factors. More work needs to be done to clarify the role of ddPCR for the identification of high-level HER2 amplification and to possibly also play a role in determination of the newly introduced HER2-low category [31].

## Data Availability

Data to support the reporting of results in Virchows Archiv can be requested from DDr. Beate Alinger-Scharinger, University Hospital (SALK) & Paracelsus Medical University Salzburg (PMU), Salzburg, Austria and by email sent electronically to b.alinger-scharinger@salk.at.
