# Correction to: HER2 copy number determination in breast cancer using the highly sensitive droplet digital PCR method

**DOI:** 10.1007/s00428-023-03716-1

**Published:** 2023-12-07

**Authors:** Beate Alinger-Scharinger, Cornelia Kronberger, Georg Hutarew, Wolfgang Hitzl, Roland Reitsamer, Frederike Klaassen‑Federspiel, Martina Hager, Thorsten Fischer, Karl Sotlar, Heidi Jaksch-Bogensperger

**Affiliations:** 1 Department of Pathology, University Hospital and Paracelsus Medical University Salzburg, Muellner Hauptstraße 48, 5020 Salzburg, Austria; 2 Research Management and Technology Transfer, Paracelsus Medical University Salzburg, Strubergasse 16, 5020 Salzburg, Austria; 3 Department of Ophthalmology and Optometry, Paracelsus Medical University Salzburg, Muellner Hauptstraße 48, 5020 Salzburg, Austria; 4 Research Program Experimental Ophthalmology and Glaucoma Research, Paracelsus Medical University Salzburg, Muellner Hauptstraße 48, 5020 Salzburg, Austria; 5 Department of Obstetrics and Gynaecology, Clinical Research Center Salzburg (CRCS), University Hospital and Paracelsus Medical University Salzburg, Muellner Hauptstraße 48, 5020 Salzburg, Austria

**Correction to: Virchows Archiv**

**https://doi.org/10.1007/s00428-023-03706-3**

In the original version of this article, the given and family names of authors were incorrectly structured. The names were displayed correctly in all versions at the time of publication.

The original article has been corrected.

